# The triple cost of tobacco and sugary drinks: health, economy and the environment

**DOI:** 10.17843/rpmesp.2025.423.15560

**Published:** 2025-09-29

**Authors:** Andrea Alcaraz, Natalia Espinola

**Affiliations:** 1 Departamento de Evaluación de Tecnologías Sanitarias y Evaluaciones Económicas, Instituto de Efectividad Clínica y Sanitaria, Buenos Aires, Argentina. Departamento de Evaluación de Tecnologías Sanitarias y Evaluaciones Económicas Instituto de Efectividad Clínica y Sanitaria Buenos Aires Argentina

## INTRODUCTION

In Latin America, the consumption of tobacco and sugary drinks is among the most relevant risk factors for public health. The region shows a high burden of preventable morbidity and mortality from chronic diseases, a significant use of health resources (hospitalizations, treatments), and great pressure on the budgets of the health system and families, disproportionately affecting the most vulnerable households. These outcomes have been widely documented in scientific literature and in reports from international organizations. 

However, this approach, focused on the health and economic dimensions, although correct, is incomplete. The analysis often leaves a critical dimension out of the picture: the environmental footprint. Tobacco and sugary drinks are mass-consumption, single-use products with a high impact in terms of waste, greenhouse gas emissions in their production and distribution chain, intensive use of water and energy, and ecosystem degradation. Cigarette butts and plastic bottles are consistently among the most commonly found waste on beaches, riverbanks, and cities worldwide. 

By analyzing these aspects separately, we underestimate the true magnitude of the problem and obtain partial diagnoses. This editorial proposes to broaden the perspective by recognizing that tobacco and sugary drinks not only make people sick and put pressure on budgets, but also deteriorate the environment; therefore, leaving out this dimension erodes the effectiveness of public policies.

## IMPACT ON HEALTH AND THE ECONOMY

The health and economic burden of tobacco and sugary drink consumption is widely documented in the region. In eight countries that account for about 80% of the population of Latin America, smoking is associated with 351,000 deaths, 2.25 million disease events, 12.2 million disability-adjusted life years (DALYs), and US$22.8 billion in direct medical costs. If the losses in labor productivity (US$16.2 billion) and the cost of informal care (US$10.8 billion) are added, the economic losses reach 1.4% of the aggregate Gross Domestic Product (GDP) ^1^. These are avoidable costs that put sustained pressure on the health budgets of the region. 

For sugary drinks, in Argentina, Brazil, El Salvador, and Trinidad and Tobago, their consumption is associated in one year with 18,000 deaths (≈3.2% of all deaths from disease), seven million disease events, about half a million Disability-Adjusted Life Years (DALYs), and US$2 billion in direct medical costs for health systems ^2^. 

The burden is inequitable: in Argentina, families in the lowest quintile bear 35.2% of their per capita income in costs attributable to smoking, compared to 5.4% in the highest quintile ^3^. The result: a double penalty, greater exposure to disease, and less ability to face medical costs.

## ENVIRONMENTAL IMPACT OF TOBACCO

Tobacco products contaminate throughout their entire life cycle: from cultivation to disposal. The impact, which is rarely integrated into the health debate, affects basic resources, climate, land, water, and air, and progresses like a cascade: curing, manufacturing, transportation, consumption, and waste. 

Life cycle estimates attribute 84 megatons of CO₂ per year to tobacco (~0.2% of global emissions), with ~14 g per cigarette. Each year, >5.3 million hectares are allocated to cultivation (an area similar to Costa Rica), with deforestation, biodiversity loss, intensive pesticide use, and soil acidification from curing, competing with food production. In water, the tobacco cycle contaminates or depletes >22 billion m³ annually, and one cigarette can affect an average of 3.7 liters. In the air, in addition to the pollutants from smoke for smokers and non-smokers, combustion releases large quantities of toxic substances: an estimated 138-451 million kg of tar in five years. At the end of the chain are the butts: of ~6 trillion cigarettes consumed per year, ~4.5 trillion are discarded into the environment. They are the most ubiquitous waste globally; in 2024, Ocean Conservancy reported that in Latin America they were the second most found item on beaches (after plastic beverage bags) ^5^. 

The footprint is also not neutral in terms of global justice: the leaf and products are mostly exported from low- and middle-income countries that, in net terms, absorb the environmental costs of others’ consumption, deepening inequities. In parallel, the industry “greenwashes” processes and packaging without modifying the structural damage. And the evidence on the environmental impact of electronic cigarettes and heated tobacco products, still consolidating, already anticipates a large-scale problem: electronic waste, lithium batteries, plastic capsules, and new chains of contamination ^4^.

## ENVIRONMENTAL IMPACT OF SUGARY DRINKS

Sugary drinks also impose a high environmental cost, especially due to single-use plastic packaging and the water used in their production. Life cycle analyses show that manufacturing and disposing of the packaging generate much more impact than producing or transporting the drink. In a sugary carbonated beverage, the packaging accounts for ~60-80% of the environmental impact per liter; keeping it cold at the point of sale can increase the carbon footprint by up to 33%, while transportation contributes <7% ^6^.

The water footprint (the amount of water required for production) of a 500 ml plastic bottle of sugary drink is estimated to be 169-309 liters, with almost all of this associated with the supply chain, particularly sugar ^7^. 

The massive disposal of single-use containers is connected to the waste crisis in Latin America. The UN notes that disposable plastic bottles are consistently among the most found objects on beaches and that the environmental impact per liter of beverage (e.g., kg CO₂e/L, liters of water/L, and waste/L) depends on the type of container and its end-of-life; reuse and return systems, and higher rates and content of recycling, clearly improve it ^8^. 

Even so, significant gaps persist: an extensive coastal strip and populations near the sea, along with still-developing management infrastructures, facilitate plastic leakage into rivers and oceans. In 2020, there were 3.7 million tons of plastic waste with the potential to enter the ocean in Latin America and the Caribbean, with projected increases for 2030 and 2050 if the current trend is not reversed ^9^.

### Implications for public policies

If we accept that the problem is threefold—health, economy, and environment—the response must be designed to achieve simultaneous and sustainable benefits. ‘Health taxes’ are cost-effective for reducing the consumption of tobacco and sugary drinks. Their revenue can be allocated to verifiable public goods—safe water in schools and health centers, waste infrastructure, and support for recycling cooperatives. With an explicit destination, legitimacy grows, and the policy is sustained. 

The second axis is regulatory. For filters and packaging, apply extended producer responsibility, return and reuse targets, recycled content standards, and clear labeling about end-of-life, thereby avoiding the transfer of environmental costs to municipalities and households. In parallel, 100% smoke-free environments and food environments that limit the supply and marketing of sugary drinks align the health signal with the environmental one: less exposure and less waste. 

None of this works if each sector operates in its own lane. A common architecture for measurement and governance is needed that integrates health, economic, and environmental indicators, with territorial disaggregation so as not to lose sight of equity. The ministries of health, environment, and education, local governments, producers, businesses, cooperatives, and civil society must agree on goals, deadlines, and responsibilities. At a regional scale, cooperation to harmonize packaging standards, fiscal criteria, and sustainable public procurement can prevent regulatory fragmentation, which currently dilutes results. 

## CONCLUSION

Tobacco and sugary drinks are not two separate debates: they form part of the same chain of impacts that sickens, puts pressure on budgets, and degrades soil, water, and air. Keeping them in separate tracks produces incomplete diagnoses and partial policies. What is needed is a common framework that articulates public health and sustainability, with shared goals, comparable indicators, traceable financing, and clear responsibilities at all links in the chain. 

An integrated approach enables the prioritization of interventions with a triple benefit: reduced disease and mortality, lower avoidable spending for families and systems, and lower waste and emissions. The invitation is to build that framework in a spirit of cooperation, among ministries, local governments, producers, businesses, schools, cooperatives, and civil society, and with open accountability. 

It is not about adding isolated measures, but about organizing a shared strategy. If we do so, the result will be seen in daily life: fewer butts on the beach, fewer bottles in the river, and fewer beds occupied by preventable causes. The challenge is complex, but the opportunity is historic: to advance towards policies that simultaneously protect the health of people and the health of the planet.
