# Supplementary material for: The triple cost of tobacco and sugary drinks: health, economy and the environment
Source: Rev Peru Med Exp Salud Publica. 2025 Sep 29;42(3):237–9. doi: 10.17843/rpmesp.2025.423.15560 (PMC12679973; doi:10.17843/rpmesp.2025.423.15560)
Supplement: Translation

# Translation: El triple costo del tabaco y bebidas azucaradas: salud, economía y ambiente

**Authors:** Andrea Alcaraz, Natalia Espinola

**Affiliations:** 1 Departamento de Evaluación de Tecnologías Sanitarias y Evaluaciones Económicas, Instituto de Efectividad Clínica y Sanitaria, Buenos Aires, Argentina.

## INTRODUCCIÓN

En América Latina, el consumo de tabaco y de bebidas azucaradas se ubica entre los factores de riesgo más relevantes para la salud pública. La región evidencia una alta carga de morbimortalidad evitable en enfermedades crónicas, un significativo uso de recursos sanitarios (internaciones, tratamientos) y una gran presión sobre los presupuestos del sistema de salud y de las familias; afectando de manera desproporcionada a los hogares más vulnerables. Estos resultados han sido ampliamente documentados en la literatura científica y en informes de organismos internacionales.

Sin embargo, este enfoque, centrado en las dimensiones sanitaria y económica, aunque correcto, resulta incompleto. El análisis suele dejar fuera de cuadro una dimensión crítica: la huella ambiental. El tabaco y las bebidas azucaradas son productos de consumo masivo, de un solo uso y con un elevado impacto en residuos, emisiones de gases de efecto invernadero en su cadena de producción y distribución, uso intensivo de agua y energía, y degradación de ecosistemas. Las colillas de cigarrillos y las botellas plásticas figuran sistemáticamente entre los desechos más encontrados en playas, riberas y ciudades del mundo.

Al analizar por separado estos planos, subestimamos la magnitud real del problema y obtenemos diagnósticos parciales. Esta editorial propone ampliar la mirada reconociendo que el tabaco y las bebidas azucaradas no solo enferman personas y ejercen presión sobre presupuestos, sino que también deterioran el medio ambiente; por lo que dejar fuera esa dimensión erosiona la efectividad de las políticas públicas.

## IMPACTO EN LA SALUD Y LA ECONOMÍA

La carga sanitaria y económica del consumo de tabaco y bebidas azucaradas está ampliamente documentada en la región. En ocho países que concentran cerca del 80% de la población de América Latina, el tabaquismo se asocia cada año con 351 000 muertes, 2,25 millones de eventos de enfermedad, 12,2 millones de años de vida ajustados por discapacidad (AVAD) y US$ 22,8 mil millones de dólares en costos médicos directos. Si a esto se suman las pérdidas de productividad laboral (US$ 16,2 mil millones) y el costo del cuidado informal (US$ 10,8 mil millones), las pérdidas económicas alcanzan el 1,4% del Producto Interno Bruto (PIB) agregado ^1^. Son costos evitables que presionan de manera sostenida a los presupuestos sanitarios de la región.

Para las bebidas azucaradas, en Argentina, Brasil, El Salvador y Trinidad y Tobago, su consumo se asocia en un año con 18 000 muertes (≈3,2% de todas las muertes por enfermedad), siete millones de eventos de enfermedad, alrededor de medio millón de Años de Vida Ajustados a Discapacidad (AVAD) y US$ 2 mil millones en costos médicos directos para los sistemas de salud ^2^.

La carga es inequitativa: en Argentina, las familias del quintil inferior soportan un 35,2% de su ingreso per cápita por costos atribuibles al tabaquismo, frente al 5,4% del quintil superior ^3^. El resultado: doble penalidad, mayor exposición a la enfermedad y menor capacidad de enfrentar los costos médicos.

## IMPACTO AMBIENTAL DEL TABACO

Los productos de tabaco contaminan a lo largo de todo su ciclo de vida: desde el cultivo hasta el descarte. El impacto, que rara vez se integra al debate sanitario, atraviesa recursos básicos, clima, tierra, agua y aire, y avanza como una cascada: curado, manufactura, transporte, consumo y residuos.

Las estimaciones de ciclo de vida atribuyen al tabaco 84 megatoneladas de CO₂ por año (~0,2% de las emisiones globales), con ~14 g por cigarrillo. Cada año se destinan >5,3 millones de hectáreas al cultivo (una superficie similar a Costa Rica), con deforestación, pérdida de biodiversidad, uso intensivo de plaguicidas y acidificación de suelos por el curado, compitiendo con la producción de alimentos. En agua, el ciclo del tabaco contamina o agota >22 mil millones de m³ anuales y un cigarrillo puede afectar en promedio 3,7 litros. En aire, además de los contaminantes del humo para fumadores y no fumadores, la combustión libera grandes cantidades de sustancias tóxicas: se estiman 138-451 millones de kg de alquitrán en cinco años. Al final de la cadena quedan las colillas: de ~6 billones de cigarrillos consumidos por año, ~4,5 billones se arrojan al ambiente. Son el residuo más ubicuo a nivel global; en 2024, Ocean Conservancy reportó que en América Latina fueron el segundo ítem más hallado en playas (tras las bolsas de bebidas plásticas) ^5^.

La huella tampoco es neutra en términos de justicia global: la hoja y los productos se exportan mayormente desde países de ingresos bajos y medianos que absorben, en neto, los costos ambientales del consumo ajeno, profundizando inequidades. En paralelo, la industria “pinta de verde” procesos y empaques sin modificar el daño estructural. Y la evidencia sobre el impacto ambiental de cigarrillos electrónicos y dispositivos de tabaco calentado, todavía en consolidación, ya anticipa un problema de gran escala: residuos electrónicos, baterías de litio, cápsulas plásticas y nuevas cadenas de contaminación ^4^.

## IMPACTO AMBIENTAL DE LAS BEBIDAS AZUCARADAS

Las bebidas azucaradas también imponen un alto costo ambiental, sobre todo por los envases plásticos de un solo uso y por el agua empleada en su producción. Los análisis de ciclo de vida muestran que fabricar y desechar el envase genera mucho más impacto que elaborar o transportar la bebida. En una bebida gaseosa azucarada, el packaging explica ~60-80% del impacto ambiental por litro; mantenerla fría en el punto de venta puede aumentar la huella de carbono hasta 33%, mientras el transporte aporta <7% ^6^.

La huella hídrica (la cantidad de agua para la elaboración) de una botella plástica de bebida azucarada de 500 ml se estima en 169-309 litros, casi toda asociada a la cadena de insumos, especialmente al azúcar ^7^.

El descarte masivo de envases de un solo uso se conecta con la crisis de residuos en América Latina. La ONU recuerda que las botellas plásticas desechables figuran sistemáticamente entre los objetos más hallados en playas y que el impacto ambiental por cada litro de bebida (p. ej., kg CO₂e/L, litros de agua/L y residuos/L) depende del tipo de envase y de su fin de vida; los sistemas de reutilización y retorno, y mayores tasas y contenido de reciclado, lo mejoran claramente ^8^.

Aun así, persisten grandes brechas: una extensa franja costera y poblaciones cercanas al mar, junto con infraestructuras de gestión aún en desarrollo, facilitan fugas de plástico hacia ríos y océanos. En 2020 hubo 3,7 millones de toneladas de desechos plásticos con potencial de entrar al océano en América Latina y el Caribe, con aumentos proyectados para 2030 y 2050 si no se revierte la tendencia ^9^.

### Implicancias para políticas públicas

Si aceptamos que el problema es triple, salud, economía y ambiente, la respuesta debe diseñarse para obtener beneficios simultáneos y sostenibles. Los ‘impuestos saludables’ son costo-efectivos para reducir el consumo de tabaco y bebidas azucaradas. Su recaudación puede destinarse a bienes públicos verificables, agua segura en escuelas y centros de salud, infraestructura de residuos, apoyo a cooperativas de reciclaje, con un destino explícito, crece la legitimidad y la política se sostiene.

El segundo eje es regulatorio. Para filtros y envases, aplicar responsabilidad extendida del productor; metas de retorno y reutilización; estándares de contenido reciclado; y etiquetado claro sobre el fin de vida, evitando trasladar costos ambientales a municipios y hogares. En paralelo, ambientes 100% libres de humo y entornos alimentarios que limiten oferta y marketing de bebidas azucaradas alinean la señal sanitaria con la ambiental: menos exposición y menos residuos.

Nada de esto funciona si cada sector corre por su carril. Hace falta una arquitectura común de medición y gobernanza que integre indicadores sanitarios, económicos y ambientales, con desagregación territorial para no perder de vista la equidad. Los ministerios de salud, ambiente y educación, gobiernos locales, productores, comercios, cooperativas y sociedad civil deben acordar metas, plazos y responsabilidades. Y a escala regional, la cooperación para armonizar estándares de envases, criterios fiscales y compras públicas sostenibles puede evitar la fragmentación normativa que hoy diluye los resultados.

## CONCLUSIÓN

El tabaco y las bebidas azucaradas no son dos debates separados: forman una misma cadena de impactos que enferma, ejerce presión sobre presupuestos y degrada suelo, agua y aire. Mantenerlos en carriles distintos produce diagnósticos incompletos y políticas parciales. Lo que hace falta es un marco común que articule salud pública y sostenibilidad, con metas compartidas, indicadores comparables, financiamiento trazable y responsabilidades claras en todos los eslabones de la cadena.

Un enfoque integrado permite priorizar intervenciones con triple beneficio: menos enfermedad y mortalidad, menor gasto evitable para las familias y los sistemas, y menos residuos y emisiones. La invitación es a construir ese marco en clave de cooperación, entre ministerios, gobiernos locales, productores, comercios, escuelas, cooperativas y sociedad civil, y con rendición de cuentas abierta.

No se trata de sumar medidas sueltas, sino de organizar una estrategia compartida. Si lo hacemos, el resultado se verá en la vida cotidiana: menos colillas en la playa, menos botellas en el río y menos camas ocupadas por causas prevenibles. El desafío es complejo, pero la oportunidad es histórica: avanzar hacia políticas que protejan simultáneamente la salud de las personas y la salud del planeta.
